# Discordant amyloid-β PET and CSF biomarkers and its clinical consequences

**DOI:** 10.1186/s13195-019-0532-x

**Published:** 2019-09-12

**Authors:** Arno de Wilde, Juhan Reimand, Charlotte E. Teunissen, Marissa Zwan, Albert D. Windhorst, Ronald Boellaard, Wiesje M. van der Flier, Philip Scheltens, Bart N. M. van Berckel, Femke Bouwman, Rik Ossenkoppele

**Affiliations:** 1 Department of Neurology & Alzheimer Center, Amsterdam Neuroscience, Vrije Universiteit Amsterdam, Amsterdam UMC, P.O. Box 7057, 1007 MB Amsterdam, The Netherlands; 2 Department of Health Technologies, Tallinn University of Technology, Tallinn, Estonia; 3 Center of Radiology, North Estonia Medical Centre, Tallinn, Estonia; 4 Neurochemistry laboratory, Department of Clinical Chemistry, Amsterdam Neuroscience, Vrije Universiteit Amsterdam, Amsterdam UMC, Amsterdam, The Netherlands; 5 Department of Radiology & Nuclear Medicine, Amsterdam Neuroscience, Vrije Universiteit Amsterdam, Amsterdam UMC, Amsterdam, The Netherlands; 6 Department of Epidemiology & Biostatistics, Amsterdam Neuroscience, Vrije Universiteit Amsterdam, Amsterdam UMC, Amsterdam, The Netherlands; 7 Clinical Memory Research Unit, Lund University, Malmö, Sweden

**Keywords:** Subjective cognitive decline, Mild cognitive impairment, Dementia, Alzheimer’s disease, Positron emission tomography, Cerebrospinal fluid, Amyloid

## Abstract

**Background:**

In vivo, high cerebral amyloid-β load has been associated with (i) reduced concentrations of Aβ_42_ in cerebrospinal fluid and (ii) increased retention using amyloid-β positron emission tomography. Although these two amyloid-β biomarkers generally show good correspondence, ~ 10–20% of cases have discordant results. To assess the consequences of having discordant amyloid-β PET and CSF biomarkers on clinical features, biomarkers, and longitudinal cognitive trajectories.

**Methods:**

We included 768 patients (194 with subjective cognitive decline (SCD), 127 mild cognitive impairment (MCI), 309 Alzheimer’s dementia (AD), and 138 non-AD) who were categorized as concordant-negative (*n* = 315, 41%), discordant (*n* = 97, 13%), or concordant-positive (*n* = 356, 46%) based on CSF and PET results. We compared discordant with both concordant-negative and concordant-positive groups on demographics, clinical syndrome, apolipoprotein E (*APOE*) ε4 status, CSF tau, and clinical and neuropsychological progression.

**Results:**

We found an increase from concordant-negative to discordant to concordant-positive in rates of *APOE* ε4 (28%, 55%, 70%, *Z* = − 10.6, *P* < 0.001), CSF total tau (25%, 45%, 78%, *Z* = − 13.7, *P* < 0.001), and phosphorylated tau (28%, 43%, 80%, *Z* = − 13.7, *P* < 0.001) positivity. In patients without dementia, linear mixed models showed that Mini-Mental State Examination and memory composite scores did not differ between concordant-negative (*β* [SE] − 0.13[0.08], *P* = 0.09) and discordant (*β* 0.08[0.15], *P* = 0.15) patients (*P*_interaction_ = 0.19), while these scores declined in concordant-positive (*β* − 0.75[0.08] patients (*P*_interaction_ < 0.001). In patients with dementia, longitudinal cognitive scores were not affected by amyloid-β biomarker concordance or discordance. Clinical progression rates from SCD to MCI or dementia (*P* = 0.01) and from MCI to dementia (*P* = 0.003) increased from concordant-negative to discordant to concordant-positive.

**Conclusions:**

Discordant cases were intermediate to concordant-negative and concordant-positive patients in terms of genetic (*APOE* ε4) and CSF (tau) markers of AD. While biomarker agreement did not impact cognition in patients with dementia, discordant biomarkers are not benign in patients without dementia given their higher risk of clinical progression.

**Electronic supplementary material:**

The online version of this article (10.1186/s13195-019-0532-x) contains supplementary material, which is available to authorized users.

## Introduction

Alzheimer’s disease (AD) is the most common cause of dementia and is characterized by accumulation of amyloid-β (Aβ) plaques in the earliest phase of the disease [1, 2]. There are currently two established methods for detecting presence of Aβ pathology in vivo, i.e., reduced concentrations of Aβ 1–42 (Aβ_42_) in CSF and increased retention of Aβ PET tracers [3, 4]. These biomarkers have been incorporated in research and diagnostic criteria [5–8].

Within these criteria, it is assumed that CSF Aβ_42_ and Aβ PET can be used interchangeably, based on mounting evidence showing strong associations between binary or continuous PET and CSF biomarkers [9–14]. Nonetheless, 10–20% of study participants have discordant results (i.e., CSF+/PET− or CSF−/PET+). Discordance in Aβ PET and CSF biomarkers potentially has important ramifications for their application in clinical, investigational, or trial settings. A glimpse of this was provided by a previous study assessing longitudinal differences in cognition between participants without dementia with different CSF/PET profiles [15]. They found no memory decline in concordant-negative (CSF−/PET−) and discordant (CSF+/PET−) groups, while the concordant-positive (CSF+/PET+) group did deteriorate over time.

In the current study, we compared discordant (CSF+/PET− and CSF−/PET+) with concordant-negative (CSF−/PET−) and concordant-positive (CSF+/PET+) patients across four diagnostic groups (subjective cognitive decline (SCD), mild cognitive impairment (MCI), AD dementia, and non-AD dementia) in terms of (i) baseline demographics, cognition, *APOE* ε4 status, and CSF tau levels, (ii) longitudinal cognitive trajectories, and (iii) changes in clinical diagnosis.

## Materials and methods

### Study population

We included 768 patients who visited our tertiary memory clinic between November 2005 and November 2017 and underwent both lumbar puncture and Aβ PET within 365 days. All patients underwent a standard diagnostic evaluation consisting of medical history, informant-based history, neurological examinations, neuropsychological testing, basic laboratory testing, apolipoprotein E (*APOE*) genotyping, MRI, and CSF [16]. Clinical diagnoses at baseline were established by consensus at multidisciplinary meetings using conventional diagnostic criteria, without knowledge of CSF results. Aβ PET was not part of standard diagnostic evaluation and was performed separately within the context of clinical research studies. Clinical follow-up including neuropsychological examination was performed annually. CSF and Aβ PET results were available to clinicians at time of follow-up visits. Patients were divided into four diagnostic groups: SCD, MCI, AD, and non-AD. SCD refers to patients presenting with cognitive complaints in the absence of objective cognitive decline or neurologic impairment (i.e., criteria for MCI, dementia, or any neurologic or psychiatric disorder not met). Patients with a syndrome diagnosis of dementia and a suspected non-AD etiology were categorized as non-AD dementia (e.g., frontotemporal dementia, vascular dementia, dementia with Lewy bodies or progressive supranuclear palsy). Patients with a postponed or other neurological diagnosis (69 (9%) at baseline and 48 (6%) after their last visit) were included in one of the four diagnostic groups based on the probable syndrome diagnosis and suspected etiology, as indicated by the neurologist in the medical records. The closest visit with a full neuropsychological assessment within a year of the first Aβ biomarker test was considered the baseline visit.

### Neuropsychological assessment

Cognitive functioning was assessed using a standardized neuropsychological test battery covering global cognition and five cognitive domains (i.e., memory, language, attention, executive, and visuospatial functions) [17]. For global cognition, we used the Mini-Mental State Examination (MMSE). We used the Visual Association Test (VAT) and total immediate recall and delayed recall of the Dutch Version of the Rey Auditory Verbal Learning Test for memory. For language, we used the VAT naming and category fluency (animals). For attention, we used the Trail Making Test (TMT) part A, the forward condition of the Digit Span, and the Stroop Test card I (word) and II (color). We used the TMT part B, the backward condition of the Digit Span, Stroop Test card III (word-color), Frontal Assessment Battery, and the Dutch version of the Controlled Oral Word Association Test (letter fluency) for executive functioning. Finally, we assessed visuospatial functioning using three subsets of the Visual Object and Space Perception (VSOP) battery: (i) incomplete letters, (ii) dot counting, and (iii) number location.

Neuropsychological data were transformed to *Z*-scores, using the mean and standard deviations of 360 cognitively normal individuals (mean age ± SD 58 ± 8, female sex 140 (39%)), who were cerebrospinal fluid biomarker-negative and visited our memory clinic between 2001 and 2015 [18]. TMT A, TMT B, and the Stroop Tests were log transformed to account for their non-normal distribution, and inverted by computing − 1 × *Z*-score, so that lower scores indicate worse test performance. When TMT B was aborted during the task (328/1986 (17%) observations), we estimated the TMT B by multiplying the time needed to complete the TMT A with the mean TMT B/A ratio from the respective diagnostic group. For the five cognitive domains, we calculated mean *Z*-scores by averaging all completed tests in each domain. A domain *Z*-score was generated if a patient had completed a minimum of one test per domain. The proportions of missing neuropsychological test results are shown in Additional file 2: Table S1. At least one follow-up visit was available for 538 (70.0%) patients. The median follow-up time was 1.9 (IQR 1.1–2.7) years.

### CSF

We obtained CSF by a lumbar puncture between L3/4, L4/5, or L5/S1 intervertebral space, using a 25-gauge needle and a syringe [16]. We collected the samples in polypropylene microtubes, centrifuged at 1800*g* for 10 min at 4 °C. Thereafter, the samples were frozen at − 20 °C until manual analysis of Aβ_42_, total tau, and tau phosphorylated at threonine 181 (p-tau) using sandwich ELISAs [Innotest assays: β-amyloid 1–42, tTAU-Ag, and PhosphoTAU-181p; Fujirebio (formerly Innogenetics)] at the Neurochemistry laboratory of the Department of Clinical Chemistry of VUmc. As the median CSF Aβ_42_ values of our cohort have been gradually increasing over the years, we corrected all Aβ_42_ values to adjust for the longitudinal upward drift [16]. In short, based on the cross section of bimodal distributions of Aβ_42_ concentrations in our memory clinic cohort, year-specific cut points were determined with Gaussian mixture modeling. By this approach, every Aβ_42_ value in the total Amsterdam Dementia Cohort was retrospectively modified to adjust for the drift, allowing to use a uniform Aβ_42_ cut-off value of < 813 pg/mL. This method was validated using three different approaches, of which one was by calculating its concordance with amyloid PET results (88%). Cut-off values for total tau and p-tau were > 375 pg/mL and > 52 pg/mL respectively [19].

### PET

Aβ PET is not routine in our diagnostic work-up but is usually performed as part of research programs or sometimes as an add-on diagnostic test [16]. We performed Aβ PET on either the Gemini TF PET-CT, Ingenuity TF PET-CT, Ingenuity PET/MRI system (all Philips Medical Systems, Best, The Netherlands), and ECAT EXACT HR+ scanner (Siemens Healthcare, Erlangen, Germany) PET scanners. We included 271 (35%) patients who underwent PET using [^11^C]PIB, 24 (3%) using [^18^F]florbetapir, 151 (20%) using [^18^F]flutemetamol, and 322 (42%) using [^18^F]florbetaben. All acquisition and processing procedures have been described in detail elsewhere [20–25]. For all PET scans, whole-brain visual assessment was performed by an experienced nuclear medicine physician (BvB), according to guidelines approved by the FDA ([^18^F]florbetapir, [^18^F]flutemetamol, and [^18^F]florbetaben) or as described previously ([^11^C]PIB) [21, 24, 25]. Scans were rated as positive or negative for the presence of Aβ pathology. Aβ PET scans were performed within a median of 54 (IQR 14–75) days of the lumbar puncture.

### Classification of patients

Based on CSF Aβ_42_ and Aβ PET results, patients were categorized into three groups: concordant-negative (PET−/CSF−), discordant (combined CSF+/PET− or CSF−/PET+), or concordant-positive (CSF+/PET+).

### Statistical analysis

Statistical analysis was performed using R software (Version 3.4.3, The R Foundation for Statistical Computing). We compared baseline demographic, clinical, and cognitive characteristics between discordant and concordant (both negative and positive) patients within each diagnostic group, and used chi-squared tests, two samples *t*-tests, and Wilcoxon rank-sum tests where appropriate. We calculated the overall concordance rate between Aβ PET and CSF Aβ_42_ as a percentage of concordant patients of the whole study population. To validate the concordance rate, we performed receiver operating characteristic (ROC) analysis to calculate the area under the curve (AUC) of the CSF total tau/Aβ_42_ ratio for amyloid PET positivity. Note that we used the drift-adjusted CSF Aβ_42_ values, but the original CSF tau values, as the drift in time only pertained to measurements of Aβ_42_ [26]. We defined the cut-point (0.44) that maximized the Youden index for amyloid PET positivity and calculated diagnostic accuracy [27]. We used chi-squared tests to assess differences in proportions of discordance between the different Aβ PET tracers. To examine trends for increased proportions of *APOE* ε4 carriership, levels of CSF total tau and p-tau, and diagnostic conversion (both progression and regression) from concordant-negative to discordant to concordant-positive, we used the Cochrane-Armitage trend test [28]. For these analyses, we dichotomized levels of CSF total tau and p-tau for consistency (see “CSF” section).

We used linear mixed models to assess changes in domain-specific neuropsychological *Z*-scores and MMSE scores over time, stratifying for patients with and without dementia, comparing discordant patients with both concordant-negative and concordant-positive groups. We used a random intercept with a fixed slope, and adjusted for age, sex, and education. The models further included terms for time and CSF/PET profiles, as well as an interaction term time × CSF/PET profiles. Data are presented as *β* coefficients (SE), reflecting annual change in composite *Z*-scores. The *P* value for slope represents the significance of the interaction between time and group, separately analyzed within groups (concordant-negative, discordant, and concordant-positive). The *P* value for interaction represents the significance of the interaction between time and concordant-negative and concordant-positive groups with the discordant group as reference. We performed Bonferroni correction for group-wise testing on all comparisons between concordant and discordant groups and applied a significance level of *P* < 0.05.

### Standard protocol approvals, registrations, and patient consent

The institutional review board of the VU University Medical Center approved all individual studies from which the current data was gathered and retrospectively analyzed. All patients provided written informed consent for their data to be used for research purposes [20–25].

### Data availability statement

All published and unpublished anonymized data from this study can be made available upon reasonable request from a qualified investigator to the corresponding author.

## Results

### Discordance between Aβ CSF and PET

**Fig. 1:**
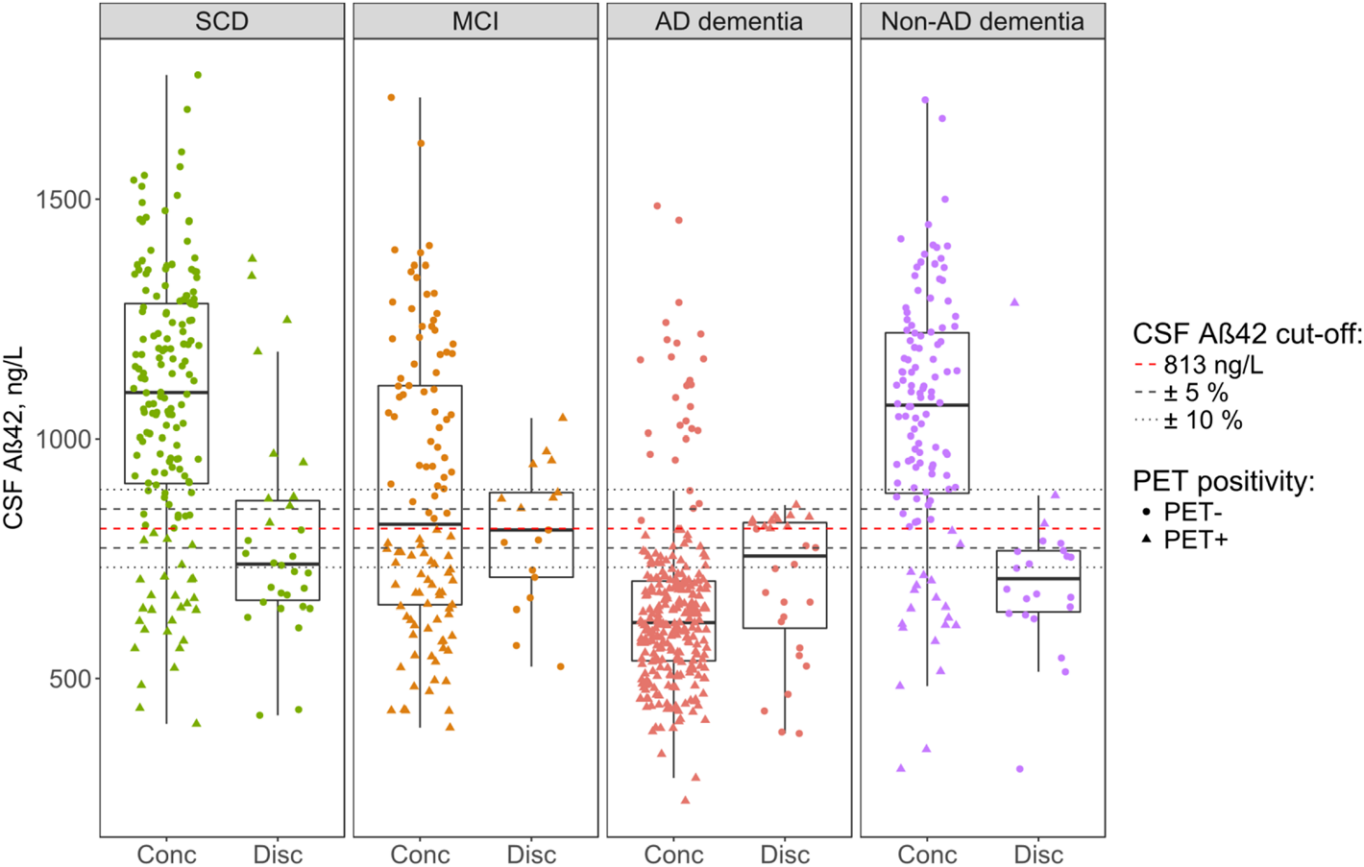
Distribution of CSF Aβ_42_ CSF/PET discordant and concordant patients per syndrome diagnosis. Abbreviations: *AD*, Alzheimer’s disease; *CSF*, cerebrospinal fluid; *Conc*, concordant; *Disc*, discordant; *MCI*, mild cognitive impairment; *SCD*, subjective cognitive decline

Across all groups, discordance between CSF and PET was *n* = 97 (13%). When discordant, CSF was more often positive than PET (67% vs. 33%, *P* < 0.001). The proportion of patients with a discordant CSF/PET profile varied between diagnostic groups, but was not significantly different (SCD 15%, MCI 13%, AD dementia 9%, and non-AD dementia 16%, *P* = 0.13) (Table 1). When excluding patients with a CSF value within 5% (range 773–853 pg/mL) or 10% of the cut-off value (range 732–894 pg/mL), the overall discordance decreased from 13 to 11 to 9% respectively. This indicates that accounting for threshold issues lowers biomarker discrepancies, but concordance remained at a similar level. The decrease in discordance was most prominent in patients with AD dementia (from 9 to 5 to 5%, Table 1 and Fig. 1). We also examined PET-CSF discordance using the CSF total tau/Aβ_42_ ratio, using a cut-off derived from predicting amyloid PET positivity. Similarly, this resulted in overall 13% PET-CSF discordance (14% in SCD, 9% in MCI, 9% in AD dementia and 24% in non-AD dementia).

**Table 1:** Rate of discordance across diagnostic groups

	Total	SCD	MCI	AD dementia	Non-AD dementia
*N* (%)	768	194 (25)	127 (17)	309 (40)	138 (18)
Discordant, cut-off < 813 ng/L (%)*	97 (13)	30 (15)	17 (13)	28 (9)	22 (16)
*CSF+/PET− (%)*	65 (67)	20 (67)	9 (53)	17 (61)	19 (86)
Discordant, excl. ± 5% cut-off (%)	75 (11)	27 (15)	14 (12)	15 (5)	19 (15)
Discordant, excl. ± 10% cut-off (%)	56 (9)	20 (12)	10 (10)	13 (5)	13 (11)

*Abbreviations*: *AD* Alzheimer’s disease, *CSF* cerebrospinal fluid, *MCI* mild cognitive impairment, *PET* positron emission tomography, *SCD* subjective cognitive decline

*Proportion of discordant patients between diagnostic groups does not differ significantly (chi-squared test)

The proportion of patients with a discordant CSF/PET profile across the different Aβ PET tracers varied between 9 and 17% but was not significantly different (*P* = 0.53) (Additional file 1: Figure S1). A total of 47/768 (6%) had repeated (≥ 2) amyloid PET scans, of which 28 patients were scanned using different tracers. Amyloid PET result changed in only 3 patients over time, going from negative to a positive result.

### Baseline characteristics

Of all patients (*n* = 768), 194 (29%) had SCD, 127 (17%) MCI, 309 (40%) AD dementia, and 138 (18%) non-AD types of dementia (Table 2). The non-AD type dementia group included the frontotemporal dementia spectrum (66, 48%), dementia with Lewy bodies (22, 16%), vascular dementia (6, 4%), and other dementia syndromes (44, 32%) like progressive supranuclear palsy and corticobasal syndrome. Overall, discordant patients did not differ in age, sex, and education from concordant-negative and concordant-positive patients. At baseline, discordant patients had lower scores for MMSE and the cognitive domains memory, language, and visuospatial than concordant-negative patients. In contrast, discordant patients performed better on MMSE and the memory domain than concordant-positive patients.

**Table 2:** Baseline demographic and clinical characteristics

CSF/PET profile	Total (*N* = 768)			SCD (*N* = 194)			MCI (*N* = 127)			AD dementia (*N* = 309)			Non-AD dementia (*N* = 138)		
	−/−	Disc.	+/+	−/−	Disc.	+/+	−/−	Disc.	+/+	−/−	Disc.	+/+	−/−	Disc.	+/+
*N* (%)	315 (41)	97 (13)	356 (46)	136 (70)	30 (15)	28 (14)	55 (43)	17 (13)	55 (43)	28 (9)	28 (9)	253 (82)	96 (70)	22 (16)	20 (14)
Age (SD)	63 (8)	63 (9)	64 (7)	60 (7)	60 (7)	61 (9)	67 (7)	66 (9)	64 (8)	65 (7)	65 (8)	63 (7)	64 (8)	63 (9)	67 (5)
Sex, male (%)	211 (67)	58 (60)	192 (54)	85 (63)	20 (67)	11 (39)	43 (78)	10 (59)	32 (58)	20 (71)	13 (46)	136 (54)	63 (66)	15 (68)	13 (65)
Education (IQR)	5 (4–6)	5 (4–6)	5 (4–6)	6 (5–6)	5 (4–6)	6 (5–7)	6 (5–6)	6 (5–6)	5 (5–6)	5 (4–6)	5 (4–6)	5 (4–6)	5 (4–5)	5 (4–5)	6 (5–6)
MMSE (SD)	26 (3)^c^	24 (4)	23 (4)^b^	28 (2)	27 (3)	28 (3)	27 (2)	26 (3)	27 (2)	24 (3)	22 (4)	22 (4)	24 (4)	23 (5)	24 (4)
Cognitive domains (*Z*-scores):															
Memory (SD)	−1.4 (2.3)^c^	−2.5 (2.9)	−3.3 (2.8)^a^	− 0.3 (0.9)^a^	−0.9 (1.7)	−0.3 (1.0)	−1.6 (2.0)	−2.1 (1.8)	−2.3 (1.8)	−3.4 (2.3)	−4.0 (3.5)	−4.0 (2.8)	−2.3 (2.9)	−3.0 (3.1)	−2.3 (2.1)
Language (SD)	−0.7 (1.3)^b^	−1.3 (2.1)	−1.0 (1.8)	−0.1 (0.8)	−0.2 (0.5)	0.0 (0.5)	−0.5 (0.7)	−0.8 (0.8)	−0.2 (0.4)^b^	−1.3 (1.3)	−1.9 (2.2)	−1.3 (1.9)	−1.4 (1.7)	−2.3 (3.0)	−2.0 (2.9)
Attention (SD)	−0.7 (1.1)	−0.9 (1.0)	−1.1 (1.2)	−0.2 (0.8)	−0.5 (1.0)	−0.2 (1.3)	−0.5 (0.8)	−0.6 (1.0)	−0.3 (0.7)	−1.2 (1.1)	−1.3 (0.9)	−1.4 (1.2)	−1.4 (1.2)	−1.4 (1.0)	−1.4 (1.0)
Executive (SD)	−1.0 (1.4)	−1.3 (1.4)	−1.5 (1.4)	−0.2 (1.0)	−0.5 (1.3)	−0.1 (1.0)	−0.8 (0.9)	−0.6 (0.9)	−0.5 (0.9)	−1.9 (1.1)	−2.1 (1.1)	−1.9 (1.3)	−2.1 (1.3)	−1.9 (1.4)	−1.9 (1.3)
Visuospatial (SD)	−0.3 (1.2)^a^	−0.9 (1.8)	−1.4 (2.4)	0.0 (0.6)	−0.4 (1.8)	0.0 (1.0)	−0.3 (1.0)	−0.7 (1.1)	−0.1 (1.0)	−0.8 (1.3)	−1.4 (2.1)	−1.8 (2.6)	−0.8 (1.6)	−1.2 (1.5)	−1.2 (1.4)

*Abbreviations*: *AD* Alzheimer’s disease, *CSF* cerebrospinal fluid, *IQR* interquartile range, *MCI* mild cognitive impairment, *PET* positron emission tomography, *SCD* subjective cognitive decline, *SD* standard deviation

Data are presented as No. (%), mean (SD) or median (IQR). Within diagnostic groups, we calculated differences between discordant and both concordant groups

Education was unavailable for 28 (4%) patients, APOE genotype for 32 (4%), and MMSE for 15 (2%). Based on missing data, we could not construct a *Z*-score for *n* (%) patients for the following domains: 41 (5%) for memory, 48 (6%) for language, 43 (6%) for attention, 21 (3%) for executive functioning, and 67 (9%) for visuospatial functioning

^a^*P* < 0.05

^b^*P* < 0.01

^c^*P* < 0.001

### *APOE* ε4, CSF total tau, and p-tau levels

**Fig. 2:**
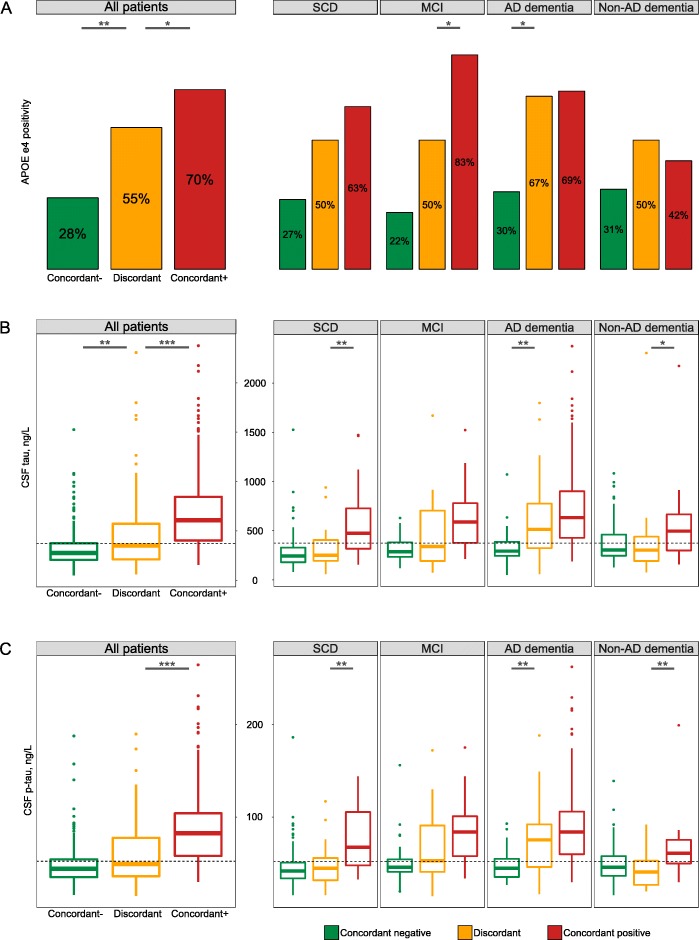
Differences in **a** *APOE* ε4 genotype, **b** CSF total tau, and **c** phosphorylated tau levels between discordant and concordant patients. Abbreviations: *AD*, Alzheimer’s disease; *APOE*, apolipoprotein E; *CSF*, cerebrospinal fluid; *MCI*, mild cognitive impairment; *SCD*, subjective cognitive decline. Dotted lines on boxplot graphs represent clinical cut-offs for CSF total tau (375 ng/L) and phosphorylated tau (52 ng/L). Significance levels for group comparisons: *P < 0.05; **P < 0.01, ***P < 0.001

Figure 2a shows the distribution of *APOE* ε4 status in discordant and concordant patients across the whole sample, as well as its distribution within the different diagnostic groups. Trend analyses showed that there is an increase of the proportion of *APOE* ε4 positivity from concordant-negative to discordant to concordant-positive, across the whole sample (Cochrane-Armitage trend test *Z*-score = − 10.6). *APOE* ε4 positivity was comparable between CSF+/PET− and CSF−/PET+ groups (52% versus 60%. *P* = 0.65). Similarly, *APOE* ε4 positivity was not a significant predictor (*P* = 0.49) in a logistic regression model involving only the CSF/PET discordant population (*n* = 97) with discordant group status (either CSF+/PET− or CSF−/PET+) as the outcome. There was a similar trend within SCD (*Z* = − 3.9), MCI (*Z* = − 6.4), and AD dementia (*Z* = − 3.8) (all *P* < 0.001), but not in the non-AD group (*Z* = − 1.3, *P* = 0.18). Analyses for dichotomized CSF total tau (cut-off > 375 pg/mL) (Fig. 2b) and CSF p-tau (cut-off > 52 pg/mL) (Fig. 2c) showed the same trend across the whole sample (total tau: *Z* = − 13.7, p-tau: *Z* = − 13.6) and within SCD (total tau: *Z* = − 5.5, p-tau: *Z* = − 3.9), MCI (total tau: *Z* = − 5.0, p-tau: *Z* = − 5.6), and AD dementia (total tau: *Z* = − 5.6, p-tau: *Z* = − 6.1) (all *P* < 0.001), as discordant patients had higher CSF total tau and p-tau levels than concordant-negative patients, while concordant-positive patients had higher CSF total tau and p-tau levels than discordant patients.

### Longitudinal cognitive trajectories

**Fig. 3:**
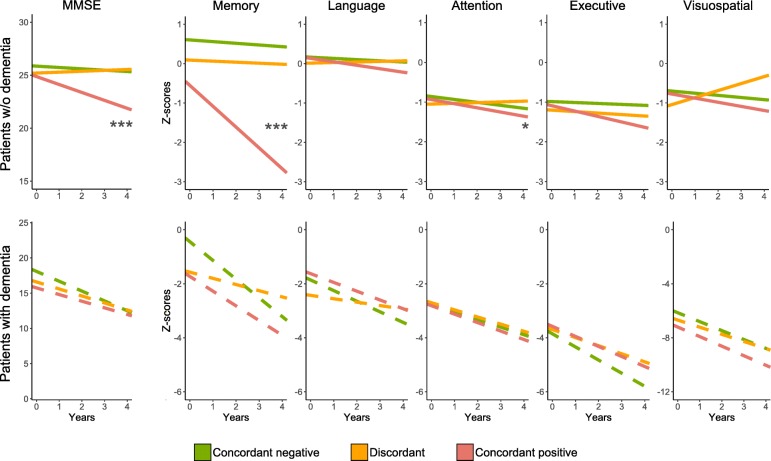
Cognitive trajectories of patients without and with dementia based on discordance and concordance. 0–5% of data points for MMSE and 0–2% of data points for *Z*-scores (memory, language, attention, executive, visuospatial) lie outside of the time range visualized on graphs. Significance levels for group comparisons: ***P < 0.001

Next, we performed linear mixed models to examine cognitive changes over time. Results are presented for the non-dementia (SCD and MCI combined) and dementia (combined AD and non-AD dementia) groups (Fig. 3 and Additional file 3: Table S2). In the non-dementia group, there was no difference in MMSE score over time between discordant (*β* 0.08 [0.15]; *P* for slope = 0.56) and concordant-negative patients (*β* − 0.13, [0.08]; *P* for slope = 0.09; *P* for interaction = 0.19), while discordant patients performed better than concordant-positive patients (*β* [SE] − 0.75, [0.08]; *P* for slope < 0.001; *P* for interaction < 0.001). Results for longitudinal decline in memory function were similar, as discordant (*β* − 0.03 [0.09]; *P* for slope = 0.78) and concordant-negative patients (*β* − 0.04 [0.05]; *P* for slope = 0.38, *P* for interaction = 0.87) did not differ, while discordant patients demonstrated less decline than concordant-positive patients (*β* − 0.53 [0.05]; *P* for slope < 0.001; *P* for interaction = < 0.001). In addition, discordant patients (*β* 0.02 [0.04]; *P* for slope = 0.68) had better attention scores over time than concordant-positive patients (*β* − 0.10 [0.03]; *P* for slope < 0.001; *P* for interaction = 0.02). There were no group differences in the remaining domains (i.e., language, executive, and visuospatial). In patients with dementia, the rates of cognitive decline as measured by MMSE and composite *Z*-scores of the five cognitive domains did not differ between concordant or discordant groups.

### Impact of biomarker concordance on changes in clinical diagnosis during follow-up

**Fig. 4:**
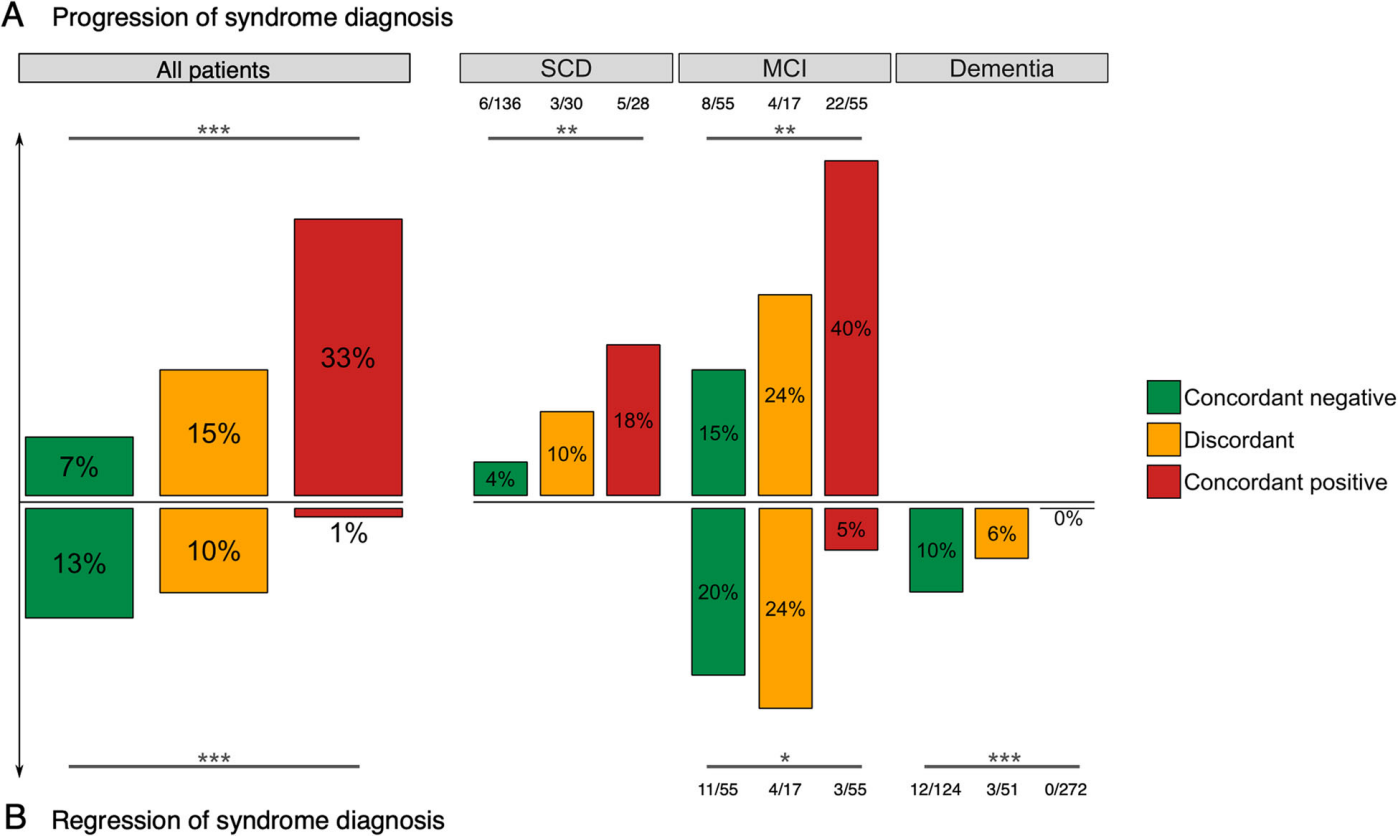
**a**, **b** Differences in change of syndrome diagnosis between discordant and concordant patients. Abbreviations: *MCI*, mild cognitive impairment; *SCD*, subjective cognitive decline. Significance levels for testing for trend: **P < 0.01, ***P < 0.001

The frequency of change in syndrome diagnosis, from SCD to MCI or dementia (*Z* = − 2.6, *P* = 0.01) and from MCI to dementia (*Z* = − 3.0, *P* < 0.01), increased with the addition of a positive Aβ marker (i.e., from concordant-negative to discordant to concordant-positive, Fig. 4a). Conversely, regression from dementia to MCI or SCD increased with the absence of a positive Aβ marker (*Z* = 5.1, *P* < 0.001), while we observed a similar trend in MCI for regression to SCD (*Z* = 2.2, *P* = 0.03 (Fig. 4b).

**Fig. 5:**
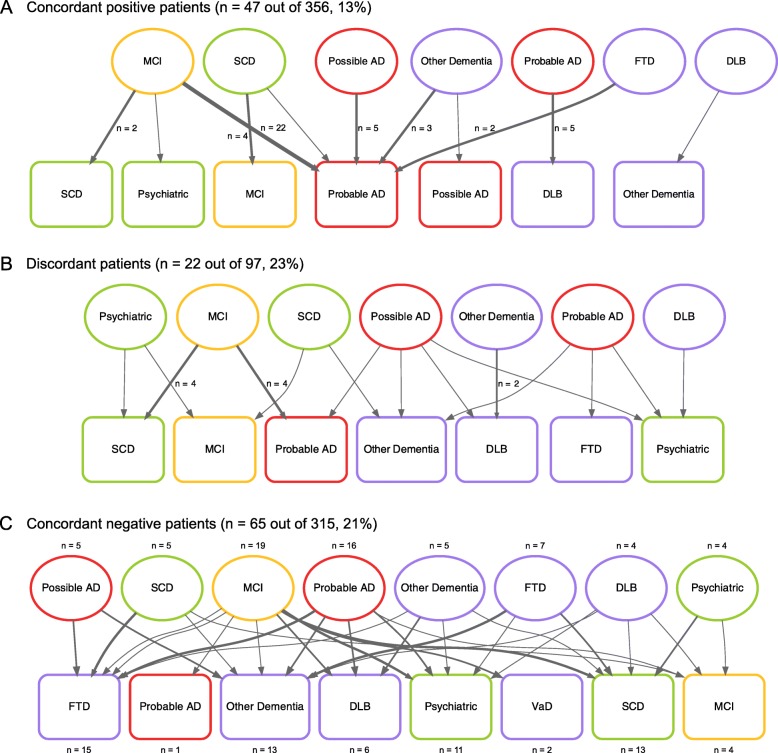
**a**–**c** Changes of clinical diagnosis during follow-up based on discordance and concordance. Abbreviations: *AD*, Alzheimer’s disease; *DLB*, dementia with Lewy bodies; *FTD*, frontotemporal dementia; *MCI*, mild cognitive impairment; *SCD*, subjective cognitive decline; *VaD*, vascular dementia

Figure 5 shows changes in clinical diagnosis, which occurred in 134 (17%) patients during a median follow-up time of 1.9 (IQR 1.1–2.7) years. These changes were similar in discordant (*n* = 22, 23%) and concordant-negative (*n* = 65, 21%) patients, but occurred less frequent in concordant-positive patients (*n* = 47, 13%) compared to discordant patients at a statistical trend level (*P* = 0.062). In discordant patients, only 5 (23%) changes were towards a diagnosis of probable AD, while the majority of changes (*n* = 36, 77%) were towards AD in concordant-positive patients. In concordant-negative patients, there was no clear pattern in the changes of clinical diagnosis. The increasing spread in distribution of diagnostic changes in patients with discordant and concordant-negative profiles suggests that the absence of a clear Aβ positive profile makes clinical decision-making less straightforward.

## Discussion

In the present study, we found that patients with discordant Aβ PET and CSF markers were intermediate to concordant-negative and concordant-positive groups on genetic (*APOE* ε4 positivity) and CSF (tau) markers of AD. In patients without dementia (SCD and MCI combined), discordant cases performed similar to concordant-negative cases in memory function and global cognition, while concordant-positive cases showed a steeper decline. Furthermore, there was an increase in the proportion of patients demonstrating change in syndrome diagnosis (from SCD to MCI or dementia, or from MCI to dementia) from concordant-negative to discordant to concordant-positive groups. In patients with dementia (AD and non-AD syndromes combined), Aβ biomarker discordance or concordance did not affect cognitive trajectories. Altogether, our findings suggest that discordant Aβ biomarkers provide important diagnostic and prognostic information in individuals without dementia.

Aβ pathology can be reliably measured in vivo using PET or in CSF, but there exists substantial discordance between these markers when obtained in the same individuals (~ 10–20% in the literature, 13% in the current study). However, whether and how Aβ biomarker discordance affects clinical progression or diagnostic changes is currently understudied. We showed that discordant patients without dementia (SCD and MCI combined) had favorable trajectories on memory and global cognitive functions compared to concordant-positive cases, which is in line with earlier studies [15]. However, compared to concordant-negative cases, patients with discordant Aβ markers were at increased risk of diagnostic progression (from SCD to MCI or dementia, or from MCI to dementia). This indicates that although the prognosis is better than in patients with two abnormal Aβ markers, positivity on a single marker in patients without dementia is not benign.

At the dementia stage, Aβ biomarker agreement did not have an effect on cognitive changes over time, as there were no differences in slopes between the concordant and discordant groups. This suggests that the relative contribution of amyloid-β pathology to cognitive impairment is limited at more advanced disease stages [29–31] and is presumably driven by other processes including accumulation of tau pathology and cerebrovascular disease. Despite the absence of an effect on cognition, biomarker discordance does seem to affect clinical decision-making, as the proportion of changed diagnoses was higher in discordant (and concordant-negative) cases compared to concordant-positive patients. This is likely due to the awareness of clinicians that a negative Aβ biomarker (even when the other marker is positive) makes the diagnosis of AD less probable [32]. This would often require a diagnostic change when AD was the initial clinical diagnosis. In contrast, positive Aβ biomarkers in non-AD syndromes do not necessarily mandate a diagnostic change, because Aβ could be considered comorbid to a primary pathology that drives the clinical presentation [33, 34]. This study suggests simultaneous assessment of Aβ PET, and CSF biomarkers provide complementary information to clinicians in certain diagnostic (i.e., differential diagnosis in patients with dementia) and prognostic (i.e., predicting clinical progression in patients without dementia) scenarios.

Among groups, discordant cases had higher rates of CSF tau and *APOE* ε4 positivity compared to concordant-negative cases. In the SCD group, this might indicate that discordant cases are further along the disease pathway and more “AD-like” than concordant-negative cases. At this early stage when Aβ burden is still relatively low, presence of Aβ might be detected earlier by one of the modalities, leading to a discordant profile. At the MCI and especially dementia stage when clinical symptoms are expressed, however, they should have significant Aβ burden that would be detected by both modalities. Yet, there was substantial discordance, especially in non-AD types of dementia. This might be explained by (i) presence of Aβ at relatively low levels as a comorbid pathology in the non-AD group, (ii) some individuals may have low resilience against Aβ pathology and show cognitive deficits at low levels of Aβ [35], (iii) differences in Aβ morphology that hamper detection by one of the modalities [36, 37], or (iv) several methodological aspects that are discussed in the paragraph below [5–8, 38]. Discordant Aβ markers have frequently been explained by suboptimal thresholds for Aβ positivity. For example, increasing the cut-off value for CSF Aβ_42_ positivity in CSF (possibly at the expense of reduced sensitivity) can increase concordance rates between PET and CSF by tipping over cases with borderline positive results [39]. Furthermore, CSF Aβ_42_ to Aβ_40_ ratios can also improve concordance rates between CSF and PET, as this accounts for interindividual variability in Aβ production, CSF turnover, or pre-analytical influences such as absorption [10, 40, 41]. The immunoassays that are being used might also explain some variance of discordance, as newer immunoassays show improved agreement between CSF and PET [42]. On the PET side, visual read metrics and quantitative threshold approaches to determine Aβ PET positivity are affected by several factors (e.g., partial volume effects, non-specific binding, or reconstruction artifacts) that could lower their accuracy [10, 43]. Nevertheless, when we excluded cases within 5% or 10% around the CSF Aβ_42_ cut-off value of 813 ng/L, relatively high discordance rates (11% and 9%) were still observed, suggesting that only a small proportion of discordant cases are explained by threshold definitions [44]. Several alternative mechanisms have been proposed that could help explaining discordance between Aβ PET and CSF biomarkers. First, the majority of discordant cases are CSF+/ PET−, with the highest proportion of CSF+/PET− profiles observed in cognitively normal individuals [10–13, 45–47]. Consequently, it was hypothesized that Aβ accumulation may be detected earlier in CSF than by Aβ PET in preclinical AD [12, 14, 15, 46, 48]. We found a similar, non-significant, trend with the highest proportion of discordant cases in the SCD and non-AD dementia groups, who are presumably at earlier phases of (age-related or comorbid) Aβ accumulation compared to MCI and AD dementia patients. Second, isolated Aβ positivity in CSF could be caused by other conditions unrelated to AD pathophysiology, such as cerebrovascular disease, neuroinflammation or amyotrophic lateral sclerosis [15, 49, 50]. A third explanation is the presence of analytical artifacts, as CSF Aβ_42_ might adsorb onto tube surfaces, which decreases available Aβ_42_ for analysis [51], while PET may yield false positive results in patients with cerebral amyloid angiopathy and false negative results in patients with atypical forms of Aβ pathology.

Strengths of this monocenter study include the large sample size with both Aβ PET and CSF data in a clinically relevant memory clinic population and the availability of longitudinal cognitive and clinical data. There are also several limitations. First and foremost, the retrospective study design (data were collected between November 2005 and November 2017) could have led to several sources of bias that we could not account for. Second, despite the large sample size, the discordant group was relatively small (*n* = 97), especially when considering that these patients were distributed across four different diagnostic groups. Within the discordant group, we therefore did not assess differences between PET+/CSF− versus PET−/CSF+ cases due to lack of statistical power. Third, we used four different Aβ PET tracers with slightly different binding properties. Although there seems to be good correspondence between Aβ PET tracers and discordant rates with CSF were within distant range (between 9 and 17%), some tracer-specific effects cannot be excluded [52]. Also, the use of different tracers complicated quantification of PET images, thus Aβ status was solely determined using a binary visual read (following procedures approved by the FDA and EMA). As such, there are no established semiquantitative scales or quantitative thresholds available for our cohort, and we were not able to analyze the frequency and characteristics of borderline PET-positive patients. Fourth, we were not able to analyze whether the previously established CSF ratio of tau to amyloid changed discordance patterns [27]. Due to the correction of Aβ_42_ values, to adjust for the longitudinal upward drift observed in our cohort and to use a uniform cut-off value, we applied a different Aβ_42_ cut-off value than previously reported [19, 27]. Fifth, amyloid PET visual reads were performed by a single experienced nuclear medicine physician, and we did not specifically examine the reproducibility of these reads. However, in a recent study assessing visual agreement of [^18^F]flutemetamol PET scans in standardized uptake value ratio (SUVr) and non-displaceable binding potential images (BP_ND)_, the nuclear medicine physician demonstrated good inter-reader agreement with a moderately experienced reader SUVr image and good intra-reader agreement between SUVr and BP_ND_ images [53]. In addition, the agreement between the SUVr and classification (positive/negative) based on quantification was good. Another study assessed inter-reader and inter-method agreement between three readers using method agreement between three readers using [^11^C]PIB PET [54]. SUVr images were visually assessed and inter-reader agreement was moderate. Finally, clinical follow-up time was relatively short, and longer follow-up is needed to further characterize the cognitive trajectories of discordant and concordant patients.

### Future directions

This study needs to be replicated in an independent sample. Such a study would preferentially be of sufficient size to be able to differentiate PET+/CSF− from PET−/CSF+, include a single Aβ PET tracer to allow PET quantification, and take a uniform approach to handling and analyzing CSF data. Furthermore, identifying the neuroimaging signature (e.g., patterns of gray matter atrophy on structural MRI or glucose hypometabolism on [^18^F]FDG PET) and neuropathological features of the discordant group could provide insight into the neurobiological mechanisms of Aβ biomarker discrepancies and AD neuropathogenesis.

## Conclusions

In conclusion, we found that patients with discordant Aβ PET and CSF markers were intermediate to concordant-negative and concordant-positive patients in terms of genetic and CSF markers of AD. Discordant biomarkers are not benign in patients without dementia given their higher risk of clinical progression, suggesting that discordant Aβ biomarkers provide important diagnostic and prognostic information in these patients.

## Additional files

Additional file 1: **Figure S1.** Proportions of discordant and concordant patients per Aβ PET tracer. Abbreviations: *AD*, Alzheimer’s disease; *CSF*, cerebrospinal fluid; *MCI*, mild cognitive impairment; *SCD*, subjective cognitive decline. (JPG 206 kb)

Additional file 2: **Table S1.** Proportions of missing neuropsychological test data per domain. (DOCX 17 kb)

Additional file 3: **Table S2.** Longitudinal slopes of cognitive domains in concordant and discordant patients. (DOCX 25 kb)

## Data Availability

The data used in this study are not publicly available but may be provided upon reasonable request.

## Abbreviations

AD: Alzheimer’s disease
APOE: Apolipoprotein E
Aβ: Amyloid-β
Aβ42: Aβ 1–42
CSF: Cerebrospinal fluid
MCI: Mild cognitive impairment
MMSE: Mini-Mental State Examination
PET: Positron emission tomography
SCD: Subjective cognitive decline
TMT: Trail Making Test
VAT: Visual Association Test

## References

[1] Masters CL, Bateman R, Blennow K, Rowe CC, Sperling RA, Cummings JL (2015). Alzheimer’s disease. Nat Rev Dis Prim.

[2] Scheltens P, Blennow K, Breteler MM, de Strooper B, Frisoni GB, Salloway S (2016). Alzheimer’s disease. Lancet.

[3] Mattsson N, Lönneborg A, Boccardi M, Blennow K, Hansson O, for the of Biomarkers G (2017). Clinical validity of cerebrospinal fluid Aβ42, tau, and phospho-tau as biomarkers for Alzheimer’s disease in the context of a structured 5-phase development framework. Neurobiol Aging.

[4] Laforce R, Soucy J, Sellami L, Dallaire-Théroux C, Brunet F, Bergeron D (2018). Molecular imaging in dementia: past, present, and future. Alzheimers Dementia.

[5] McKhann GM, Knopman DS, Chertkow H, Hyman BT, Jack CR, Kawas CH (2011). The diagnosis of dementia due to Alzheimer’s disease: recommendations from the National Institute on Aging-Alzheimer’s Association workgroups on diagnostic guidelines for Alzheimer’s disease. Alzheimers Dement.

[6] Albert MS, DeKosky ST, Dickson D, Dubois B, Feldman HH, Fox NC (2011). The diagnosis of mild cognitive impairment due to Alzheimer’s disease: recommendations from the National Institute on Aging-Alzheimer’s association workgroups on diagnostic guidelines for Alzheimer’s disease. Alzheimers Dementia.

[7] Jack CR, Bennett DA, Blennow K, Carrillo MC, Dunn B, Haeberlein S (2018). NIA-AA research framework: toward a biological definition of Alzheimer’s disease. Alzheimers Dement.

[8] Dubois B, Feldman HH, Jacova C, Hampel H, Molinuevo J, Blennow K (2014). Advancing research diagnostic criteria for Alzheimer’s disease: the IWG-2 criteria. Lancet Neurol.

[9] Schipke CG, Koglin N, Bullich S, Joachim L, Haas B, Seibyl J (2017). Correlation of florbetaben PET imaging and the amyloid peptide Aß42 in cerebrospinal fluid. Psychiatry Res Neuroimaging.

[10] Leuzy A, Chiotis K, Hasselbalch SG, Rinne JO, de Mendonça A, Otto M (2016). Pittsburgh compound B imaging and cerebrospinal fluid amyloid-β in a multicentre European memory clinic study. Brain..

[11] Palmqvist S, Zetterberg H, Mattsson N, Johansson P, Initiative A, Minthon L (2015). Detailed comparison of amyloid PET and CSF biomarkers for identifying early Alzheimer disease. Neurology..

[12] Mattsson N, Insel PS, Donohue M, Landau S, Jagust WJ (2015). Aw L, et al. independent information from cerebrospinal fluid amyloid-β and florbetapir imaging in Alzheimer’s disease. Brain..

[13] Landau SM, Lu M, Joshi AD, Pontecorvo M, Mintun MA, Trojanowski JQ (2013). Comparing positron emission tomography imaging and cerebrospinal fluid measurements of β-amyloid. Ann Neurol.

[14] Fagan AM, Mintun MA, Mach RH, Lee S, Dence CS, Shah AR (2006). Inverse relation between in vivo amyloid imaging load and cerebrospinal fluid Aβ42 in humans. Ann Neurol.

[15] Palmqvist S, Mattsson N, Hansson O, Initiative A (2016). Cerebrospinal fluid analysis detects cerebral amyloid-β accumulation earlier than positron emission tomography. Brain..

[16] van der Flier WM, Scheltens P (2018). Amsterdam dementia cohort: performing research to optimize care. J Alzheimers Dis.

[17] Leeuwis AE, Benedictus MR, Kuijer J, Binnewijzend M, Hooghiemstra AM, Verfaillie S (2017). Lower cerebral blood flow is associated with impairment in multiple cognitive domains in Alzheimer’s disease. Alzheimers Dement.

[18] Groot C, van Loenhoud AC, Barkhof F, van Berckel BN, Koene T, Teunissen CC (2017). Differential effects of cognitive reserve and brain reserve on cognition in Alzheimer disease. Neurology..

[19] Mulder C, Verwey NA, van der Flier WM, Bouwman FH, Kok A, van Elk EJ (2010). Amyloid-β (1–42), total tau, and phosphorylated tau as cerebrospinal fluid biomarkers for the diagnosis of Alzheimer disease. Clin Chem.

[20] van Berckel B, Ossenkoppele R, Tolboom N, Yaqub M, Foster-Dingley JC, Windhorst AD (2013). Longitudinal amyloid imaging using [^11^C]PIB: methodologic considerations. J Nucl Med.

[21] Ossenkoppele R, Prins ND, Pijnenburg Y, Lemstra AW, van der Flier WM, Adriaanse SF (2013). Impact of molecular imaging on the diagnostic process in a memory clinic. Alzheimers Dement.

[22] Ossenkoppele R, Tolboom N, Foster-Dingley JC, Adriaanse SF, Boellaard R, Yaqub M (2012). Longitudinal imaging of Alzheimer pathology using [^11^C]PIB, [^18^F]FDDNP and [^18^F]FDG PET. Eur J Nucl Med Mol Imaging.

[23] Ossenkoppele R, van der Flier WM, Verfaillie SC, Vrenken H, Versteeg A, van Schijndel RA (2014). Long-term effects of amyloid, hypometabolism, and atrophy on neuropsychological functions. Neurology..

[24] Zwan MD, Bouwman FH, Konijnenberg E, van der Flier WM, Lammertsma AA, Verhey FR (2016). Diagnostic impact of [^18^F]flutemetamol PET in early-onset dementia. Alzheimers Res Ther.

[25] de Wilde Arno, van der Flier Wiesje M., Pelkmans Wiesje, Bouwman Femke, Verwer Jurre, Groot Colin, van Buchem Marieke M., Zwan Marissa, Ossenkoppele Rik, Yaqub Maqsood, Kunneman Marleen, Smets Ellen M. A., Barkhof Frederik, Lammertsma Adriaan A., Stephens Andrew, van Lier Erik, Biessels Geert Jan, van Berckel Bart N., Scheltens Philip (2018). Association of Amyloid Positron Emission Tomography With Changes in Diagnosis and Patient Treatment in an Unselected Memory Clinic Cohort. JAMA Neurology.

[26] Schindler SE, Sutphen CL, Teunissen C, McCue LM, Morris JC, Holtzman DM (2018). Upward drift in cerebrospinal fluid amyloid β 42 assay values for more than 10 years. Alzheimers Dement.

[27] Duits FH, Teunissen CE, Bouwman FH, Visser P-J, Mattsson N, Zetterberg H (2014). The cerebrospinal fluid “Alzheimer profile”: Easily said, but what does it mean?. Alzheimers Dementia.

[28] Armitage P (1955). Tests for linear trends in proportions and frequencies. Biometrics..

[29] Ossenkoppele R, Jansen WJ, Rabinovici GD, Knol DL, van der Flier WM, van Berckel BN (2015). Prevalence of amyloid PET positivity in dementia syndromes: a meta-analysis. JAMA..

[30] Villemagne VL, Burnham S, Bourgeat P, Brown B, Ellis KA, Salvado O (2013). Amyloid β deposition, neurodegeneration, and cognitive decline in sporadic Alzheimer’s disease: a prospective cohort study. Lancet Neurol.

[31] Villemagne VL, Pike KE, Chételat G, Ellis KA, Mulligan RS, Bourgeat P (2011). Longitudinal assessment of Aβ and cognition in aging and Alzheimer disease. Ann Neurol.

[32] Bergeron David, Ossenkoppele Rik, Jr Laforce Robert (2018). Evidence-based Interpretation of Amyloid-β PET Results. Alzheimer Disease & Associated Disorders.

[33] Sarro L, Senjem ML, Lundt ES, Przybelski SA, Lesnick TG, Graff-Radford J (2016). Amyloid-β deposition and regional grey matter atrophy rates in dementia with Lewy bodies. Brain..

[34] Naasan G, Rabinovici GD, Ghosh P, Elofson JD, Miller BL, Coppola G (2015). Amyloid in dementia associated with familial FTLD: not an innocent bystander. Neurocase..

[35] Stern Y (2012). Cognitive reserve in ageing and Alzheimer’s disease. Lancet Neurol.

[36] Schöll M, Wall A, Thordardottir S, Ferreira D, Bogdanovic N, Långström B (2012). Low PiB PET retention in presence of pathologic CSF biomarkers in Arctic APP mutation carriers. Neurology..

[37] Yokota O, Terada S, Ishizu H, Ujike H, Ishihara T, Namba M (2003). Variability and heterogeneity in Alzheimer’s disease with cotton wool plaques: a clinicopathological study of four autopsy cases. Acta Neuropathol.

[38] Sperling RA, Aisen PS, Beckett LA, Bennett DA, Craft S, Fagan AM (2011). Toward defining the preclinical stages of Alzheimer’s disease: recommendations from the National Institute on Aging-Alzheimer’s Association workgroups on diagnostic guidelines for Alzheimer’s disease. Alzheimer’s &amp. Dementia..

[39] Zwan M, van Harten A, Ossenkoppele R, Bouwman F, Teunissen C, Adriaanse S (2014). Concordance between cerebrospinal fluid biomarkers and [^11^C]PIB PET in a memory clinic cohort. J Alzheimers Dis.

[40] Pannee J, Portelius E, Minthon L, Gobom J, Andreasson U, Zetterberg H (2016). Reference measurement procedure for CSF amyloid beta (Aβ)1-42 and the CSF Aβ1-42 /Aβ1-40 ratio - a cross-validation study against amyloid PET. J Neurochem.

[41] Willemse E, van Uffelen K, Brix B, Engelborghs S, Vanderstichele H, Teunissen C (2017). How to handle adsorption of cerebrospinal fluid amyloid β (1–42) in laboratory practice? Identifying problematic handlings and resolving the issue by use of the Aβ42/Aβ40 ratio. Alzheimers Dement.

[42] Janelidze S, Pannee J, Mikulskis A, Chiao P, Zetterberg H, Blennow K (2017). Concordance between different amyloid immunoassays and visual amyloid positron emission tomographic assessment. JAMA Neurol.

[43] Villeneuve S, Rabinovici GD, Cohn-Sheehy BI, Madison C, Ayakta N, Ghosh PM (2015). Existing Pittsburgh compound-B positron emission tomography thresholds are too high: statistical and pathological evaluation. Brain..

[44] Tijms BM, Willemse EA, Zwan MD, Mulder SD, Visser P, van Berckel BN (2017). Unbiased approach to counteract upward drift in cerebrospinal fluid amyloid-β 1–42 analysis results. Clin Chem.

[45] Cairns NJ, Ikonomovic MD, Benzinger T, Storandt M, Fagan AM, Shah AR (2009). Absence of Pittsburgh compound B detection of cerebral amyloid β in a patient with clinical, cognitive, and cerebrospinal fluid markers of Alzheimer disease: a case report. Arch Neurol.

[46] Fagan AM, Mintun MA, Shah AR, Aldea P, Roe CM, Mach RH (2009). Cerebrospinal fluid tau and ptau181 increase with cortical amyloid deposition in cognitively normal individuals: implications for future clinical trials of Alzheimer’s disease. EMBO Mol Med.

[47] Jagust W, Landau S (2009). Aw L, Trojanowski J, Koeppe R, Reiman E, et al. relationships between biomarkers in aging and dementia. Neurology..

[48] Morris E, Chalkidou A, Hammers A, Peacock J, Summers J, Keevil S (2016). Diagnostic accuracy of 18F amyloid PET tracers for the diagnosis of Alzheimer’s disease: a systematic review and meta-analysis. Eur J Nucl Med Mol Imaging.

[49] Mattsson N, Insel PS, Landau S, Jagust W, Donohue M (2014). Aw L, et al. diagnostic accuracy of CSF Ab42 and florbetapir PET for Alzheimer’s disease. Ann Clin Transl Neurol.

[50] Selnes P, Blennow K, Zetterberg H, Grambaite R, Rosengren L, Johnsen L (2010). Effects of cerebrovascular disease on amyloid precursor protein metabolites in cerebrospinal fluid. Cerebrospinal Fluid Res.

[51] Hansson Oskar, Mikulskis Alvydas, Fagan Anne M., Teunissen Charlotte, Zetterberg Henrik, Vanderstichele Hugo, Molinuevo Jose Luis, Shaw Leslie M., Vandijck Manu, Verbeek Marcel M., Savage Mary, Mattsson Niklas, Lewczuk Piotr, Batrla Richard, Rutz Sandra, Dean Robert A., Blennow Kaj (2018). The impact of preanalytical variables on measuring cerebrospinal fluid biomarkers for Alzheimer's disease diagnosis: A review. Alzheimer's & Dementia.

[52] Landau T (2014). Thurfjell L, hmidt, Margolin R, Mintun M, et al. Amyloid PET imaging in Alzheimer’s disease: a comparison of three radiotracers. European Journal of Nuclear Medicine and. Mol Imaging.

[53] Collij LE, Konijnenberg E, Reimand J, ten Kate M, den Braber A, Alves I (2019). Assessing amyloid pathology in cognitively normal subjects using [^18^F]flutemetamol PET: comparing visual reads and quantitative methods. J Nucl Med.

[54] Zwan MD, Ossenkoppele R, Tolboom N, Beunders A, Kloet RW, Adriaanse SM (2014). Comparison of simplified parametric methods for visual interpretation of 11C-Pittsburgh compound-B PET images. J Nucl Med.

