# Supplementary figures and images for: Discordant amyloid-β PET and CSF biomarkers and its clinical consequences
Source: Alzheimers Res Ther. 2019 Sep 12;11:78. doi: 10.1186/s13195-019-0532-x (PMC6739952; doi:10.1186/s13195-019-0532-x)

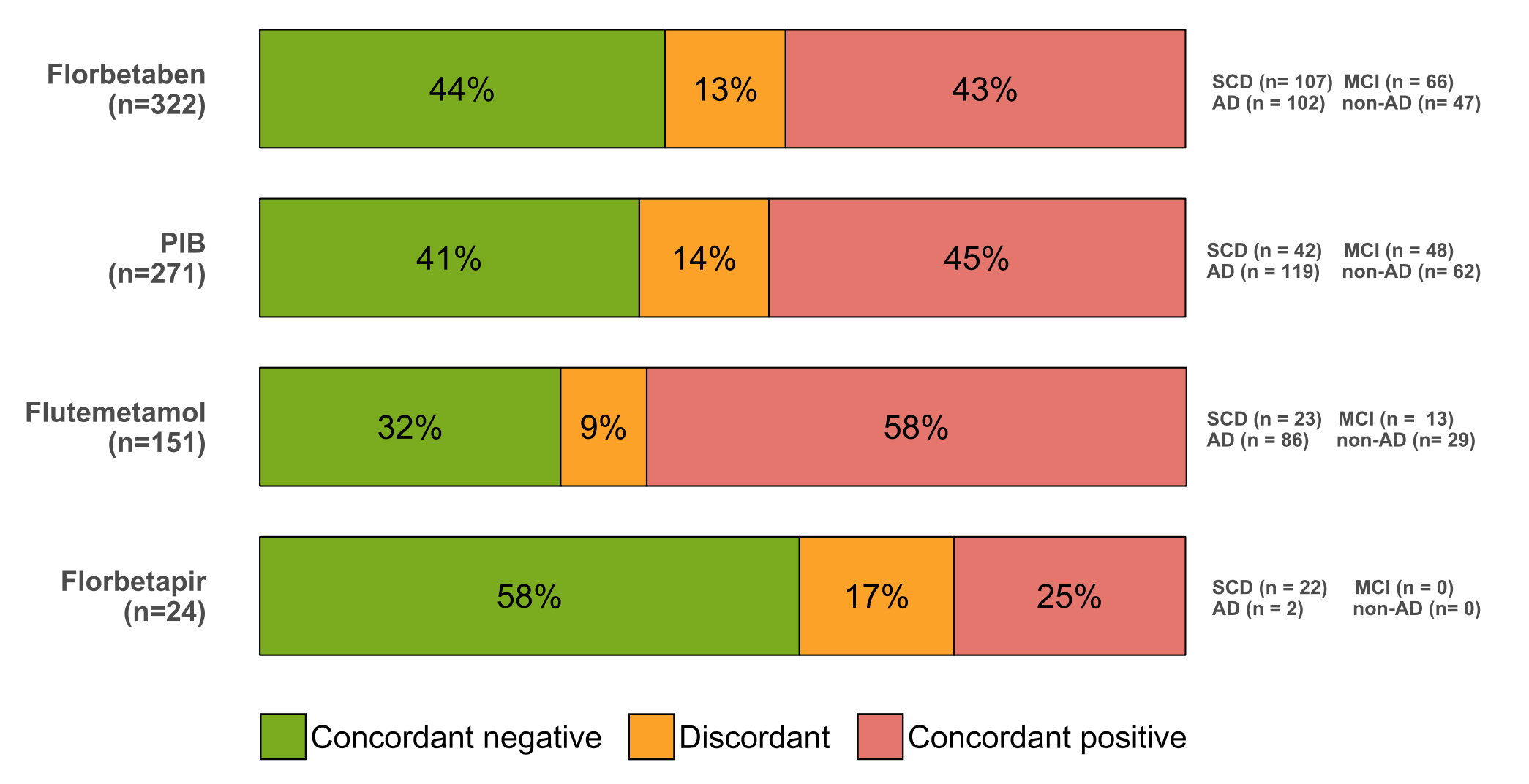

Supplement: Supplementary file 1 — Figure S1. Proportions of discordant and concordant patients per Aβ PET tracer. Abbreviations: AD, Alzheimer’s disease; CSF, cerebrospinal fluid; MCI, mild cognitive impairment; SCD, subjective cognitive decline. (JPG 206 kb) [file 13195_2019_532_MOESM1_ESM.jpg]
