# Supplementary material for: Discordant amyloid-β PET and CSF biomarkers and its clinical consequences
Source: Alzheimers Res Ther. 2019 Sep 12;11:78. doi: 10.1186/s13195-019-0532-x (PMC6739952; doi:10.1186/s13195-019-0532-x)
Supplement: Supplementary file 2 — Table S1. Proportions of missing neuropsychological test data per domain. (DOCX 17 kb) [file 13195_2019_532_MOESM2_ESM.docx]

| **Supplementary Table 1. Proportions of missing neuropsychological test data per domain** | | |  |  |  |  |
| --- | --- | --- | --- | --- | --- | --- |
| **Domain** | **Test** | **Missing** | | | | |
|  |  | **All** | **PET-/CSF-** | **Discordant** | **PET+/CSF+** | ***P* - value** |
| Global cognition | Mini-Mental State Examination | 7.0% | 7.7% | 7.6% | 6.3% | 0.486 |
| Memory | Rey Auditory Verbal Learning Test (Dutch) - Visual Association Test | 26.5% | 21.3% | 26.3% | 30.3% | <0.001 |
|  | Rey Auditory Verbal Learning Test (Dutch) - Total Immediate Recall | 24.0% | 20.5% | 21.4% | 27.0% | 0.005 |
|  | Rey Auditory Verbal Learning Test (Dutch) - Delayed recall | 24.4% | 20.9% | 21.9% | 27.4% | 0.005 |
| Language | Visual Association Test - naming | 26.6% | 21.9% | 25.4% | 30.2% | <0.001 |
|  | Category Fluency (animals) | 23.2% | 20.8% | 20.5% | 25.5% | 0.040 |
| Attention | Trail-Making Test A | 24.0% | 19.6% | 19.2% | 28.2% | <0.001 |
|  | Digit Span – Forward Condition | 26.1% | 21.7% | 24.1% | 29.6% | 0.001 |
|  | Stroop Test Card I | 34.8% | 27.9% | 34.8% | 39.9% | <0.001 |
|  | Stroop Test Card II | 36.2% | 28.7% | 35.7% | 41.7% | <0.001 |
| Executive | Trail-Making Test B | 25.8% | 20.2% | 21.0% | 30.8% | <0.001 |
|  | Digit Span – Backward Condition | 26.8% | 22.3% | 25.0% | 30.5% | 0.001 |
|  | Stroop Test Card III | 40.7% | 31.7% | 38.8% | 47.6% | <0.001 |
|  | Frontal Assessment Battery | 29.5% | 21.7% | 31.7% | 34.5% | <0.001 |
|  | Controlled Oral Word Association Test (Dutch) - Letter Fluency | 31.3% | 26.5% | 29.5% | 35.2% | <0.001 |
| Visuospatial | Visual Object and Space Perception Battery - Incomplete Letters | 39.9% | 39.0% | 40.6% | 40.4% | 0.824 |
|  | Visual Object and Space Perception Battery - Dot Counting | 39.5% | 38.3% | 42.0% | 39.8% | 0.597 |
|  | Visual Object and Space Perception Battery - Number Location | 40.2% | 37.0% | 40.6% | 42.5% | 0.064 |
