# Supplementary material for: Discordant amyloid-β PET and CSF biomarkers and its clinical consequences
Source: Alzheimers Res Ther. 2019 Sep 12;11:78. doi: 10.1186/s13195-019-0532-x (PMC6739952; doi:10.1186/s13195-019-0532-x)
Supplement: Supplementary file 3 — Table S2. Longitudinal slopes of cognitive domains in concordant and discordant patients. (DOCX 25 kb) [file 13195_2019_532_MOESM3_ESM.docx]

| **Supplementary Table 2. Longitudinal slopes of cognitive domains in concordant and discordant patients** | | | | | | | | | | | | | | |
| --- | --- | --- | --- | --- | --- | --- | --- | --- | --- | --- | --- | --- | --- | --- |
|  | Non-demented | | | | | | Demented | | | | | | | |
|  | | CSF-/PET- | p | Discordant | p | CSF+/PET+ | | p | CSF-/PET- | p | Discordant | p | CSF+/PET+ | p |
| Median (IQR) follow-up time, months | | 1.9 (1.1-2.4) |  | 1.4 (1.1-2.2) |  | 1.8 (1.1-3.0) | |  | 2.0 (1.0-2.8) |  | 1.4 (1.0-2.3) |  | 2.0 (1.1-3.0) |  |
| MMSE (SE) | | -0.13 (0.08) | 0.19 | 0.08 (0.15) | 1 | -0.75 (0.08)^b^ | | <0.001 | -1.40 (0.2) | <0.001 | -0.99 (0.27) | <0.001 | -0.94 (0.09) | <0.001 |
| Cognitive domains, Z-scores: | |  |  |  |  |  | |  |  |  |  |  |  |  |
| Memory (SE) | | -0.04 (0.05) | 0.76 | -0.03 (0.09) | 1 | -0.53 (0.05)^b^ | | <0.001 | -0.69 (0.16) | <0.001 | -0.23 (0.23) | 0.65 | -0.55 (0.09) | <0.001 |
| Language (SE) | | -0.03 (0.02) | 0.39 | 0.01 (0.04) | 1 | -0.09 (0.03) | | 0.001 | -0.39 (0.09) | <0.001 | -0.13 (0.13) | 0.66 | -0.32 (0.05) | <0.001 |
| Attention (SE) | | -0.07 (0.02) | 0.004 | 0.02 (0.04) | 1 | -0.10 (0.03)^a^ | | <0.001 | -0.28 (0.05) | <0.001 | -0.26 (0.07) | 0.001 | -0.31 (0.03) | <0.001 |
| Executive (SE) | | -0.02 (0.03) | 0.77 | -0.04 (0.05) | 0.95 | -0.14 (0.03) | | <0.001 | -0.49 (0.07) | <0.001 | -0.30 (0.11) | 0.016 | -0.37 (0.04) | <0.001 |
| Visuospatial (SE) | | -0.05 (0.04) | 0.42 | 0.18 (0.13) | 0.36 | -0.10 (0.04) | | 0.014 | -0.66 (0.12) | <0.001 | -0.53 (0.18) | 0.005 | -0.71 (0.07) | <0.001 |
| Abbreviations: IQR, interquartile range; MMSE, Mini-Mental State Examination; SE, standard error. | | | | | | | | | | | | | | |
| *P* values reported in columns indicate whether the corresponding slope was significantly different from 0. | | | | | | | | | | | | | | |
| ^a^ *P* < 0.05 | | | | | | | | | | | | | | |
| ^b^ *P* < 0.001 | | | | | | | | | | | | | | |
